# Investigation of Crystallization Growth Characteristics of Mg(OH)_2_ Crystals under Unconstrained Conditions

**DOI:** 10.3390/ma17091956

**Published:** 2024-04-23

**Authors:** Yunqing Lv, Limei Bai, Yuxin Ma, Liucheng Zhao

**Affiliations:** 1 College of Mining Engineering, North China University of Science and Technology, Tangshan 063210, China; 15350682837@163.com (Y.L.); mayuxin@ncst.edu.cn (Y.M.); zhaoliucheng@ncst.edu.cn (L.Z.); 2 Collaborative Innovation Center of Green Development and Ecological Restoration of Mineral Resources, Tangshan 063210, China; 3 Hebei Province Key Laboratory of Mining Development and Security Technology, Tangshan 063210, China

**Keywords:** high-purity magnesium hydroxide nanoparticles, crystal growth habit, hydrothermal method, the technology of environmental protection

## Abstract

Utilizing MgO as the precursor and deionized water as the solvent, this study synthesized nanoparticles of Mg(OH)_2_ via hydrothermal methods, aiming to control its purity, particle size, and morphology by understanding its growth under non-uniform nucleation. Characterization of crystal morphology and structure was conducted through scanning electron microscopy and X-ray diffraction, while laser particle size detection assessed the secondary particle size distribution. The study focused on how MgO’s hydrothermal process conditions influence Mg(OH)_2_ crystal growth, particularly through ion concentration and release rate adjustments to direct crystal growth facets. These adjustments shifted the dominant growth plane, enhancing the peak intensity ratio I001/I101 from 1.03 to 2.14, thereby reducing surface polarity and secondary aggregation of crystals. The study of the physicochemical properties of the same sample at different times revealed the pattern of crystal dissolution and recrystallization. A 2 h hydrothermal reaction notably altered the particle size distribution, with a decrease in particles sized 0.2~0.4 μm and an increase in those sized 0.4~0.6 μm, alongside new particles over 1 μm, indicating a shift toward uniformity through dissolution and recrystallization. Optimal conditions (6% magnesium oxide concentration, 160 °C, 2 h) led to the synthesis of highly dispersed, uniformly sized magnesium hydroxide, showcasing a simple, eco-friendly, and high-yield process.

## 1. Introduction

Mg(OH)_2_ is a pivotal inorganic compound with widespread applications across several domains, including wastewater treatment, insulation, flue gas desulfurization, and medicine, owing to its notable properties [1,2,3,4,5]. Distinguished from traditional halogen-based flame retardants, Mg(OH)_2_ emerges as a non-toxic, eco-friendly alternative, offering high thermal stability, smoke suppression, and environmental safety [6,7,8]. Unlike conventional halogen-based flame retardants, Mg(OH)_2_ decomposes at high temperatures to produce only MgO and H_2_O, yielding non-toxic and harmless decomposition products [9,10]. The generated H_2_O effectively absorbs heat, thus lowering the material’s surface temperature and retarding combustion [11,12]. Additionally, MgO serves as a refractory material. Compared to aluminum hydroxide, a commonly used green flame retardant, Mg(OH)_2_ exhibits a higher thermal decomposition temperature and a broader range of raw material sources [13,14,15,16].

The synthesis of magnesium hydroxide Mg(OH)_2_ primarily utilizes magnesium salts, bischofite, and active magnesium oxide, among other raw materials [17,18,19]. Jiang et al. employed magnesium chloride hexahydrate and sodium hydroxide as starting materials, utilizing urea and ethanol to tailor the morphology of Mg(OH)_2_. They synthesized the Mg(OH)_2_ through direct precipitation and subsequently aged it for 10 h, resulting in the preparation of flake-like magnesium hydroxide [20]. Liu et al. utilized bischofite as the raw material and sodium hydroxide as the precipitating agent. The Mg(OH)_2_ synthesized under atmospheric pressure was subjected to heat treatment at 200 °C for 4 h. This process yielded fine-grade magnesium hydroxide with a median particle size of 0.66 μm [21]. Huang et al. employed hydromagnesite as the raw material and followed a sequence of processes encompassing “calcination—hydration—calcination—hydrothermal” treatments. By utilizing active MgO and incorporating 4% polyvinylpyrrolidone (PVP), they synthesized Mg(OH)_2_ nanoparticles characterized by uniform morphology and excellent dispersion [22]. Wang utilized active MgO as the raw material and ethanol as a dispersing agent. Through the hydration method, he successfully prepared hexagonal flakes of high-purity Mg(OH)_2_ nanoparticles, characterized by high dispersion and uniform morphology. Utilizing active MgO as the magnesium source for the preparation of Mg(OH)_2_ through hydration results in particles with more uniform morphology and dispersion. However, the overall yield is constrained due to the stringent concentration requirements of the raw materials [23]. Further, Liu et al. conducted a hydrothermal treatment on the slurries resulting from the reaction of brine with various precipitants for 6 h. They investigated the influence of precipitants on the morphology of Mg(OH)_2_ and successfully prepared Mg(OH)_2_ with angular sheet morphology [24]. Zhao at al. subjected the Mg(OH)_2_ synthesized at ambient temperature to hydrothermal treatment for 8 h. The resulting Mg(OH)_2_ crystals exhibited distinct edges and sharp contours. In comparison to the untreated crystals, the morphology transitioned from irregular to hexagonal sheets, accompanied by significantly enhanced dispersion [25]. Additionally, Ling et al. and Wu et al. underscored the role of hydrothermal conditions in optimizing Mg(OH)_2_ particle dispersion and morphology [26,27]. Ling et al. utilized lightly fired MgO as the raw material and introduced a dispersant during water heat treatment to obtain flake Mg(OH)_2_ with excellent dispersion [26]. Wu et al. concluded from experimental analysis that increasing the hydrothermal temperature would notably enhance the dispersion of Mg(OH)_2_ particles. However, when the hydrothermal temperature exceeded 170 °C, there were no significant differences observed in particle size, morphology, or dispersion [27]. These studies collectively highlight that while hydrothermal treatment enhances Mg(OH)_2_ dispersion and uniformity, the process’s extended duration may elevate preparation costs.

The preparation of Mg(OH)_2_ through direct precipitation, using magnesium salt materials and hydromagnesite as sources, often results in poor morphological uniformity and significant agglomeration, necessitating further improvement [28]. Additionally, the generated by-products are difficult to utilize. On the other hand, using active MgO as the raw material for direct hydration can produce Mg(OH)_2_ nanoparticles with uniform morphology, monodispersity, and high purity. While this method avoids the generation of by-products, the low yield makes it challenging to meet industrial production demands. The hydrothermal method offers stable crystallization and higher yields. Nevertheless, the extended reaction time associated with this method results in higher production costs. In summary, leveraging the advantages of stable crystallization and higher yields offered by the hydrothermal method, we aim to investigate the crystalline growth behavior of Mg(OH)_2_ crystals without constraints [29,30]. By selecting appropriate crystalline growth conditions, we can control the preparation of Mg(OH)_2_ with excellent performance, thereby reducing production costs, simplifying the process, and achieving scaled production of nano-sized Mg(OH)_2_.

Accordingly, in the present article, we adopt a one-step hydrothermal approach to synthesize Mg(OH)_2_. By adjusting hydrothermal temperature, time, and solid–liquid ratio, we investigate the influence of reaction conditions on the growth of Mg(OH)_2_ crystals and the crystalline changes during the growth process. At the same time, we explore the crystalline growth characteristics of Mg(OH)_2_ crystals during hydrothermal reactions. On the basis of the green and pollution-free process route, we can stably and efficiently prepare highly dispersed and homogeneous Mg(OH)_2_ nanoparticles by controlling the main growth surface of the crystal.

## 2. Materials and Methods

### 2.1. Experimental Materials and Equipment

**Figure 1:**
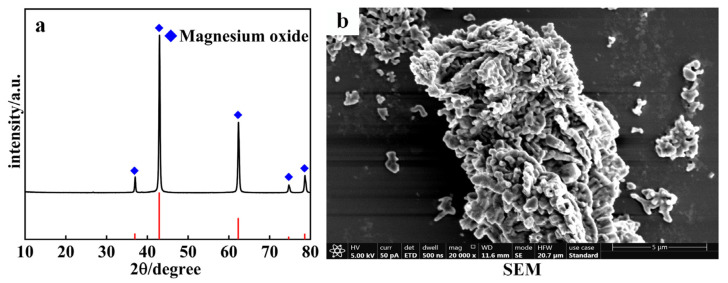
XRD pattern and microstructure image of MgO. (**a**) XRD pattern; (**b**) SEM image.

In this experiment, we utilized active MgO with a coral-like morphology and a purity of 99.2%, as shown in Figure 1b. The characteristic particle size D_50_ of the MgO was measured to be 3.39 μm, with a D_90_ of 7.46 μm. The detected high activity of this morphology of MgO was confirmed through testing. Figure 1a shows the XRD pattern of the raw MgO material. The prominent peaks correspond well to the characteristic peaks of MgO in the standard card (PDF#78-0430), indicating high purity and absence of impurity doping in the raw material used. The deionized water was prepared in-house in the laboratory. The reaction apparatus used was the reactor (GSH-2L, Weihai Global Chemical Machinery Mfg Co., Ltd., Weihai, Shandong, China).

### 2.2. Experimental Procedure

The experimental methodology implemented in this study is outlined as follows: initially, a magnetic stirring bar was positioned within a polytetrafluoroethylene (PTFE) liner, into which a specified volume of deionized water was added. This was followed by the gradual addition of a quantified amount of MgO into the liner, with concurrent stirring, to prepare the MgO slurry. The prepared slurry was then transferred into a reaction vessel, filling it to 50% capacity. Subsequently, the liner, containing the slurry, was placed inside the reaction vessel, and hydrothermal treatment was conducted under predefined parameters. Upon completion of the reaction, the vessel was cooled with running water. The product was rinsed from the interior of the vessel liner using distilled water and collected in a beaker. A double layer of medium-speed filter paper was then placed on a Buchner funnel, and the solution was slowly poured into the funnel while employing vacuum filtration. Following filtration, the product was washed three times with distilled water and anhydrous ethanol, respectively, before being dried in an oven at 105 °C for 12 h.

### 2.3. Analysis and Detection

The crystal phase and structure were analyzed using an X-ray diffractometer, with a scanning range from 10° to 80° and a scanning speed of 10°/min. The crystal size of the samples was calculated using the Scherrer Formula (1). The aggregation index was determined using Formula (2) according to the HG/T 3821-2006 standard [31].

(1) $$D=\frac{K\lambda }{\beta \cos\theta }$$

(2) $$T=\frac{D_{90}}{d}$$

Herein, *D* represents the grain size in nanometers (nm); *K* denotes the Scherrer constant, which is set at 0.89; *λ* stands for the X-ray wavelength, taken as 0.15418 nanometers (nm). *β* represents the full width at half maximum (FWHM) of the diffraction peak, with units in angstroms (Å); *θ* denotes the diffraction angle, measured in degrees (°). *T* represents the aggregation index, where *D*_90_ is the characteristic particle size of the powder, measured in nanometers (nm), and *d* represents the average particle size observed under electron microscopy, also in nanometers (nm). Microscopic morphology of the samples was observed using a focused ion beam scanning electron microscope (Scios, FEI Company, Hillsboro, OR, USA) and a field emission scanning electron microscope (S-4800, JEOL, Showima City, Tokyo, Japan). The particle size composition and distribution of the samples were analyzed using an automatic laser particle size analyzer (NKT6100-D, Beijing Haixinrui Technology Co., Ltd., Beijing, China).

## 3. Experimental Results and Discussion

### 3.1. The Influence of Reaction Temperature on the Crystalline Growth Habit of Mg(OH)_2_ Crystals

**Figure 2:**
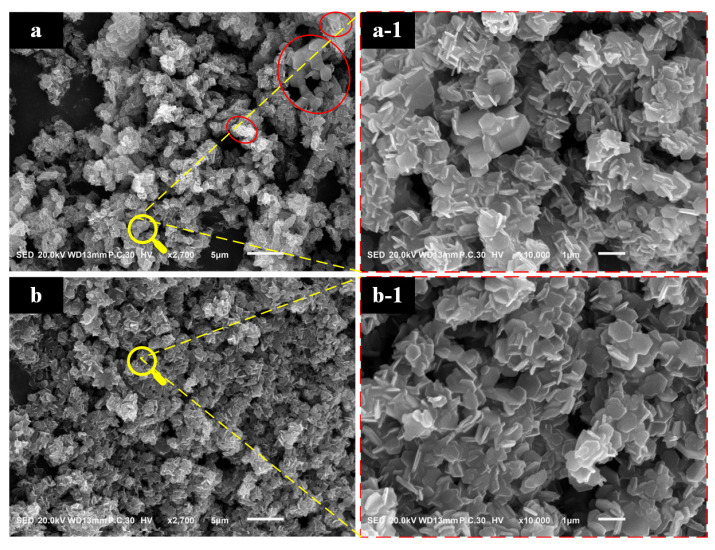
The images of magnesium hydroxide synthesized: (**a**) 120 °C; (**b**) 160 °C. (**a-1**) the magnification of (**a**), (**b-1**) the magnification of (**b**).

Figure 2 presents the morphology of Mg(OH)_2_ synthesized at various temperatures. As observed in Figure 2a, the sample contains thick plate-like Mg(OH)_2_ particles with larger diameters, where the particles exhibit indistinct boundaries. Additionally, there is significant agglomeration and intergrowth among the particles. In Figure 2b, the sample is characterized by hexagonal plate-like particles that exhibit intergrowth and agglomeration, with a more uniform particle size distribution. Notably, there are no larger particles present in this sample. Mg(OH)_2_ synthesized at both temperatures did not achieve the desired monodisperse particle distribution under optimal conditions.

Comparing the crystal morphologies in the two images, it is evident that under identical reaction times, the crystals in Figure 2b exhibit superior morphology to those in Figure 2a, with particles that are more uniform and shapes that are more regular. This indicates that the reaction temperature significantly affects the growth and crystallization of Mg(OH)_2_ crystals. Higher reaction temperatures can facilitate the dissolution of larger particles and the growth of smaller ones, which is conducive to the growth and perfection of the crystals.

**Figure 3:**
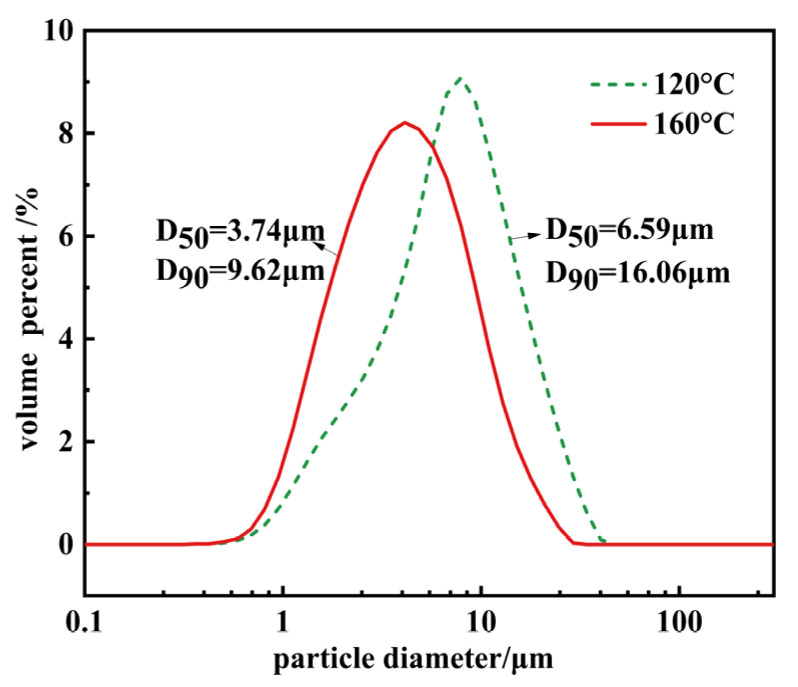
The particle size distribution patterns of Mg(OH)_2_ synthesized at different temperatures.

Figure 3 illustrates the particle size distribution graphs of Mg(OH)_2_ crystals synthesized at various temperatures. From Figure 3, it can be observed that increasing the hydrothermal reaction temperature results in the overall leftward shift of the distribution curves, with the particle size gradually approaching a normal distribution. The particle size becomes progressively more uniform. Comparing Mg(OH)_2_ particles synthesized at two different temperatures, the characteristic particle size D_50_ decreased from 6.59 μm to 3.74 μm, and D_90_ reduced from 16.06 μm to 9.62 μm upon increasing the temperature. The characteristic particle size decreased significantly. The electron microscopy average particle size, denoted as d, was calculated, based on the SEM images in Figure 2. Incorporating the characteristic particle size D_90_, the agglomeration index T decreased from 27.68 to 14.14. This decrease aligns with the patterns observed in the particle size distribution curves in Figure 3, confirming that the phenomenon of particle agglomeration has been improved.

**Figure 4:**
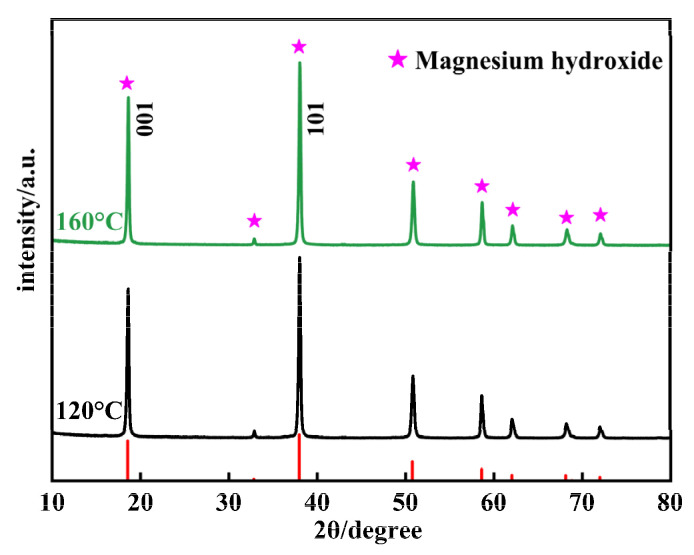
The XRD patterns of Mg(OH)_2_ synthesized at different temperatures.

Figure 4 presents the XRD patterns of Mg(OH)_2_ synthesized at varying temperatures. By comparing the prominent peaks in Figure 4 with the characteristic peaks of Mg(OH)_2_ from the standard card (PDF#84-2163), it is observed that reactive MgO can fully react to form Mg(OH)_2_ at both temperatures, with no impurity phases present. Analysis of the XRD data reveals that for the sample synthesized at 120 °C, the peak intensity for the (001) crystallographic plane is 17,870, and for the (101) crystallographic plane, it is 21,196, resulting in an I001/I101 peak intensity ratio of 0.84. For the sample synthesized at 160 °C, the peak intensity for the (001) crystallographic plane is 17,696, and for the (101) crystallographic plane it is 21,838, yielding an I001/I101 peak intensity ratio of 0.81. The peak intensities and the ratio of peak intensities (I001/I101) for the (001) and (101) crystallographic plane of the Mg(OH)_2_ crystals remain fundamentally unchanged, indicating that increasing the reaction temperature does not alter the primary growth planes during the Mg(OH)_2_ growth process. The (101) crystallographic plane continues to be the dominant exposed crystallographic plane in Mg(OH)_2_ crystals. Using the Scherrer formula to calculate the crystallite size of the planes, as the temperature increases from 120 °C to 160 °C, the crystallite size of the (001) crystallographic plane increases from 32.13 nm to 34.49 nm, and for the (101) crystallographic plane, it increases from 32.74 nm to 34.08 nm. Consequently, the average particle size grows from 32.46 nm to 34.29 nm. It is evident that increasing the reaction temperature aids in the growth of Mg(OH)_2_ crystals and simultaneously improves the agglomeration phenomenon of Mg(OH)_2_ particles. The experimental results indicate that when Mg(OH)_2_ crystals grow under unconstrained conditions, the growth rates of the (001) and (101) crystallographic plane are relatively low, while other crystallographic planes exhibit higher growth rates and less stability.

Therefore, during the hydrothermal synthesis process, as the Mg(OH)_2_ crystals undergo crystallization and growth, the (001) and (101) crystallographic planes emerge as the primary exposed crystal faces. This results in the particles’ edges appearing as inclined angles. This conclusion aligns with the simulation results of Mg(OH)_2_ crystal growth habit conducted by Fan et al. using Materials Studio 7.0 software [32].

### 3.2. The Influence of Hydrothermal Time on the Crystalline Growth Habit of Mg(OH)_2_ Crystals

**Figure 5:**
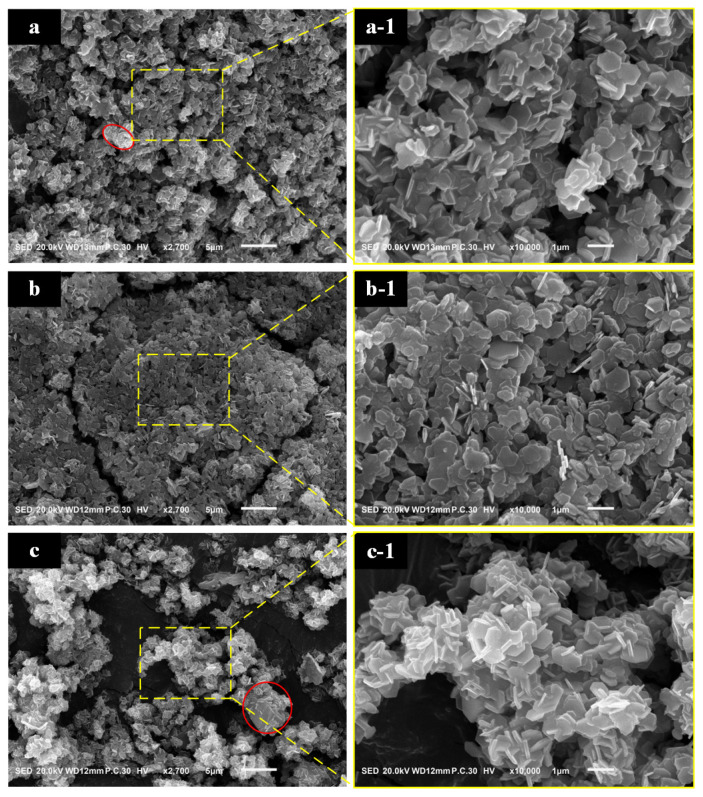
The image of Mg(OH)_2_ synthesized at different hydrothermal time: (**a**): 1 h; (**b**): 2 h; (**c**): 3 h. (**a-1**): the magnification of (**a**); (**b-1**): the magnification of (**b**); (**c-1**): the magnification of (**c**).

Figure 5 consists of SEM photographs of Mg(OH)_2_ products synthesized over varying hydrothermal durations. From Figure 5, it is observed that the sample synthesized for 2 h shows a noticeable improvement in the phenomenon of intergrowth, displaying stacked lamellar crystals, whereas the other two samples exhibit significant interspersed issues. Moreover, the sample synthesized for 3 h demonstrates the clear formation of plate-like structures, with poor uniformity in crystal growth. All three samples still present considerable agglomeration, which compromises their effective utilization.

**Figure 6:**
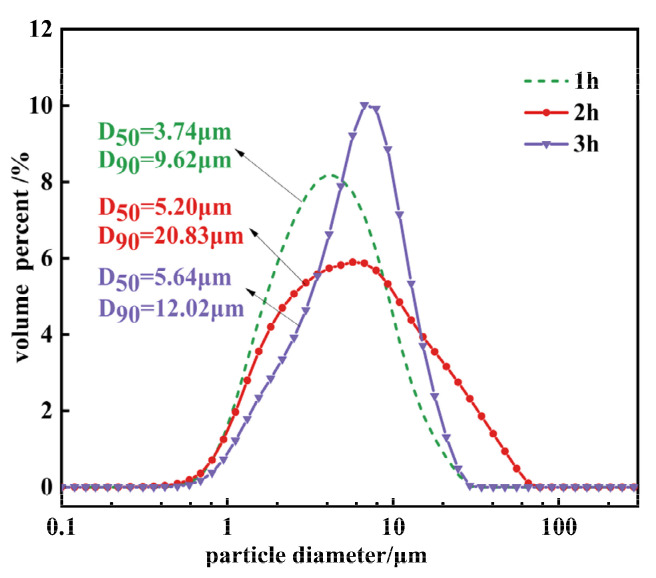
The particle size distribution of Mg(OH)_2_ synthesized at different hydrothermal time.

Figure 6 displays the particle size distribution graphs for Mg(OH)_2_ crystals synthesized over different hydrothermal durations. According to Figure 6, as the hydrothermal time increases, the characteristic particle size D_50_ of the Mg(OH)_2_ crystals increases, while the characteristic particle size D_90_ does not change in tandem. This characteristic particle size indicates that the agglomeration phenomenon of Mg(OH)_2_ crystals synthesized at all three durations is quite severe. The agglomeration index T exhibits a trend of initially increasing and then decreasing with the extension of reaction time, rising from 12.17 to 30.18 before decreasing to 17.17.

**Figure 7:**
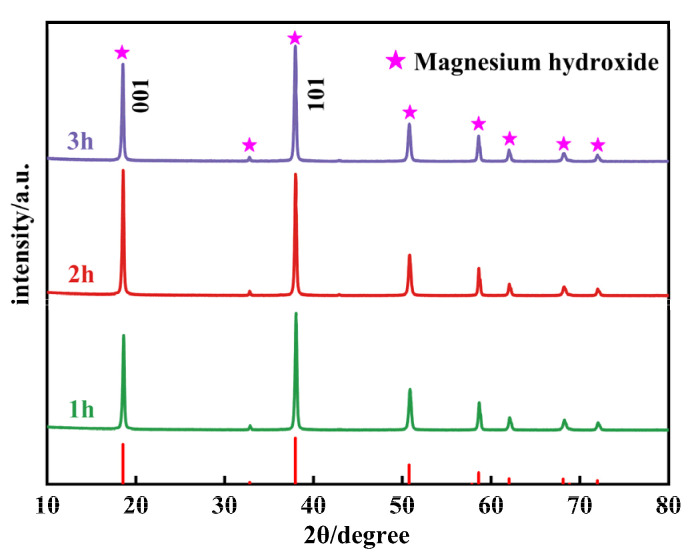
The XRD patterns of Mg(OH)_2_ synthesized at different hydrothermal time.

Figure 7 presents the XRD patterns of samples synthesized at 160 °C for various hydrothermal durations. By comparing the primary exposed peaks in Figure 7 with the characteristic peaks from the standard card (PDF#84-2163), it is confirmed that all products are Mg(OH)_2_. With the extension of hydrothermal duration, the peak intensity of the (001) crystallographic plane of Mg(OH)_2_ crystals initially increases and then decreases, while the (101) crystallographic plane shows minimal changes. This directly results in alterations to the primary exposed crystal plane and the surface polarity. For Mg(OH)_2_ crystals synthesized hydrothermally for 1 h, the peak intensity ratio of the (001) crystallographic plane to the (101) crystallographic plane is 0.81. For those synthesized for 2 h, the ratio increases to 1.03, and for 3 h, it is 0.84. Comparing these three samples, it can be observed that for Mg(OH)_2_ crystals synthesized over 2 h, the primary exposed crystal plane transitions from the (101) crystallographic plane to the (001) crystallographic plane. When the (001) crystallographic plane serves as the primary exposed surface, the surface polarity of Mg(OH)_2_ crystals decreases, leading to a reduction in the inter-crystal adsorption force, which facilitates filtration. Conversely, when the (101) crystallographic plane is the primary exposed surface, the surface polarity of the crystals is higher, resulting in stronger inter-crystal adsorption forces. This makes the particles more prone to agglomeration and more difficult to filter. Therefore, Mg(OH)_2_ prepared over 2 h exhibits weaker surface polarity compared to Mg(OH)_2_ prepared at other durations, reducing filtration time. However, according to the XRD patterns in Figure 7, although the (001) crystallographic plane becomes the primary exposed surface for the product hydrated for 2 h, its exposure is not sufficient and the polarity remains relatively high: the agglomeration phenomenon has not improved. The crystallite sizes calculated using the Scherrer formula show that the crystallite size of the (001) crystallographic plane increases from 34.49 nm to 35.72 nm and then decreases back to 34.49 nm, while the crystallite size of the (101) crystallographic plane increases from 34.08 nm to 35.84 nm and then decreases to 33.66 nm. The average particle size first increases and then decreases, indicating that the Mg(OH)_2_ crystals are in a continuous process of dissolving and recrystallizing to perfect their lattice defects.

The experimental results indicate that hydrothermal treatment can mitigate particle agglomeration, gradually improving the morphology and perfection of the crystals. During the growth process of Mg(OH)_2_, both the (001) and (101) crystallographic planes grow concurrently, and the state of crystallization continuously evolves. Consequently, given the dynamic alteration of the principal exposed crystal facets throughout the reaction process, a detailed examination of Mg(OH)_2_ crystallization at various stages is imperative for a comprehensive understanding. In the experiments, crystals grown via 2 h of hydrothermal treatment exhibited superior growth compared to Mg(OH)_2_ synthesized over other durations, with an observed improvement in the intergrowth phenomenon of Mg(OH)_2_ crystals.

### 3.3. The Influence of MgO Initial Concentration on the Crystalline Growth Habit of Mg(OH)_2_ Crystals

**Figure 8:**
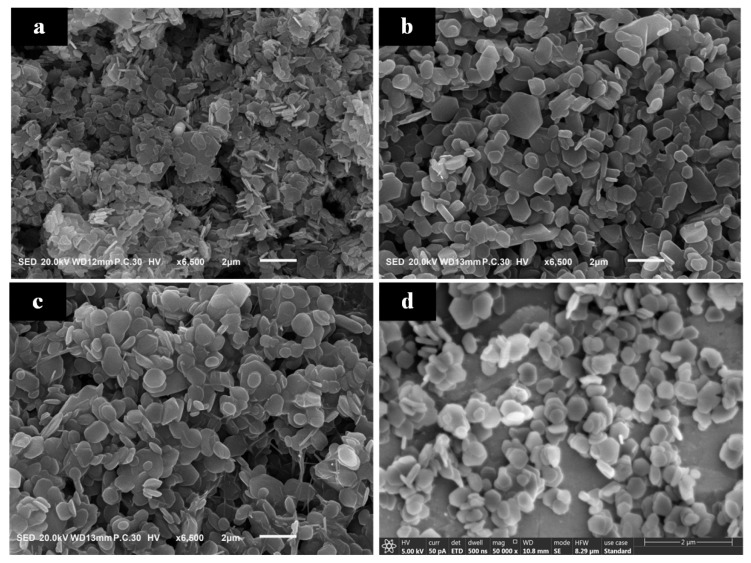
The images of Mg(OH)_2_ synthesized at different MgO initial concentration: (**a**): 2%; (**b**): 4%; (**c**): 6%; (**d**): 8%.

Figure 8 showcases SEM images of Mg(OH)_2_ crystals synthesized at varying concentrations. It is observable that in Figure 8a, the Mg(OH)_2_ crystals exhibit intergrowth with poor dispersion among particles. In contrast, Figure 8b–d show the disappearance of intergrowth phenomena, with the crystals exhibiting good growth and significantly improved dispersion.

**Figure 9:**
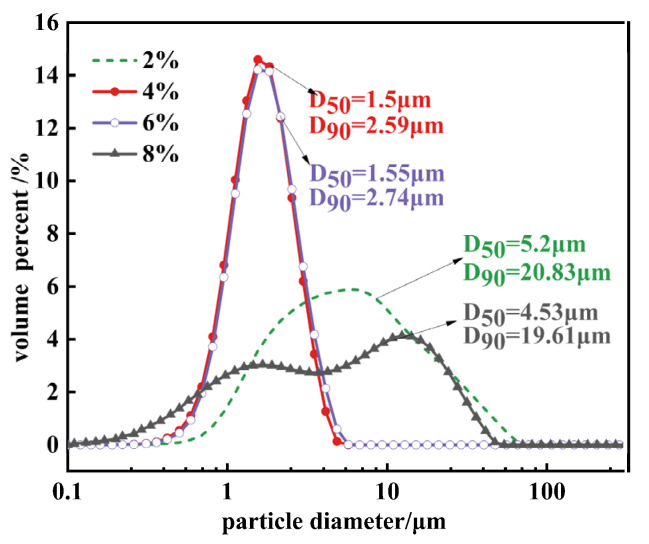
The particle size distribution of Mg(OH)_2_ synthesized at different MgO initial concentration.

Figure 9 demonstrates that particle agglomeration first improves and then worsens with changes in the initial concentration of MgO. The agglomeration index T values at the four concentrations are 27.4, 2.94, 2.58, and 46.69, respectively. It is evident that the agglomeration issue significantly improves at concentrations of 4% and 6%. When the solid-to-liquid ratio of the raw material MgO increases from 2% to 4%, there is a significant change in the particle size of Mg(OH)_2_. The characteristic particle size D_50_ decreases from 5.2 μm to 1.5 μm, and D_90_ reduces to 2.59 μm. However, further increasing the solid-to-liquid ratio of the hydrothermal raw materials, although continuing to increase the peak intensity ratio, does not significantly change the characteristic particle size. This suggests that improving the agglomeration phenomenon of Mg(OH)_2_ crystals by adjusting the amount of MgO to alter the exposure level of the (001) crystallographic plane has its limits. Continuing to increase the MgO usage could even exacerbate particle agglomeration.

**Figure 10:**
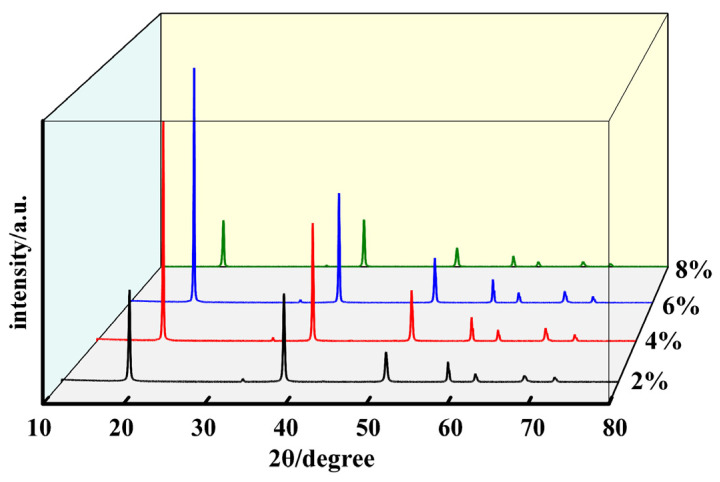
The XRD patterns of Mg(OH)_2_ synthesized at different MgO initial concentration.

Figure 10 displays the XRD patterns of Mg(OH)_2_ crystals synthesized at varying concentrations. From the figure, it is observed that as the concentration of hydrothermal raw materials increases from 2% to 6%, the (001) crystallographic plane becomes the dominant exposed crystallographic plane of Mg(OH)_2_ crystals. The peak intensity ratio of the (001) crystallographic plane to the (101) crystallographic plane gradually increases from 1.03 to 2.14, indicating a change in the crystal growth direction. Based on the theory of Mg(OH)_2_ crystal growth and the curve changes observed in Figure 9, it is understood that increasing the exposure of the (001) crystallographic plane effectively reduces the surface polarity and surface energy of the crystals, thereby ameliorating the issue of particle agglomeration. By processing the XRD data to obtain the half-peak width values and applying the Scherrer formula, the crystallite sizes of different crystallographic planes under varying concentrations are calculated. As the concentration of hydrothermal raw materials increases from 2% to 4%, the crystallite size of the (001) crystallographic plane of Mg(OH)_2_ crystals increases from 35.72 nm to 54.57 nm, and the crystallite size of the (101) crystallographic plane increases from 35.84 nm to 45.45 nm. The average particle size grows from 35.78 nm to 50.01 nm, indicating that increasing the amount of hydrothermal raw material MgO can promote the full growth of Mg(OH)_2_ particles.

By providing an ample supply of MgO, the solution is kept in a supersaturated state at room temperature. With the increase in reaction temperature, the solubility of MgO increases, leading to the hydrolysis of solid MgO and the release of Mg^2+^ ions, which promotes the hydration and crystal growth of Mg(OH)_2_. However, when increasing the MgO concentration from 4% to 6%, the characteristic particle size and crystallite sizes calculated via the Scherrer formula show almost no change. Thus, further increasing the amount of MgO does not lead to additional growth of Mg(OH)_2_ crystals but rather improves the existing crystals by reducing lattice defects, as evidenced by stronger and sharper characteristic diffraction peaks. Continuing to increase the hydrothermal raw material concentration to 8% results in the (101) crystallographic plane becoming the dominant exposed surface of Mg(OH)_2_ crystals, and a decrease in the intensity of the diffraction peaks is observed. This indicates that excessively high concentrations of MgO are not entirely beneficial for the growth of Mg(OH)_2_ crystals and can also adversely affect their growth.

The change in properties of Mg(OH)_2_ from a concentration of 6% to 8% may be attributed to the hydrolysis of active MgO in high-temperature, high-pressure aqueous solutions, releasing Mg^2+^ ions. These ions react with OH^−^ ions, dissociated from water, to form Mg(OH)_2_ octahedral growth unit ligands [28]. Given the limited availability of dissociated OH^−^ ions and the relatively slow dissociation process, an excess of Mg^2+^ ions competing for free OH^−^ ions after forming growth units leads to the attachment and stacking of Mg(OH)_2_ crystal nuclei. This competition results in slower crystal growth, poorer crystallization, and the formation of smaller particle sizes. The excess of growth units also increases the probability of crystal nucleus collisions, reducing the necessary growth time. Moreover, the diminished mass and heat transfer between a high concentration of MgO and the hydrothermal solution decrease the uniformity of the crystals, weaken the crystallization, and lead to the (101) crystallographic plane becoming the dominant exposed surface of Mg(OH)_2_ at this concentration. This surface exhibits higher polarity and is more difficult to filter. At 8% concentration, the Mg(OH)_2_ crystals have a characteristic particle size D_50_ of 4.53 μm and D_90_ of 19.61 μm. The increased surface polarity exacerbates the agglomeration phenomenon, which also accounts for the bimodal distribution observed in the particle size distribution curve. Using the Scherrer formula, the crystallite sizes for the (001) and (101) crystallographic planes are calculated to be 34.64 nm and 34.94 nm, respectively, with an average particle size of 34.79 nm. Compared to the samples at 4% and 6%, the reduction in crystal particle size demonstrates the impact of decreased mass and heat transfer on crystal growth.

### 3.4. Study on Hydrothermal Crystallization Process of Mg(OH)_2_

**Figure 11:**
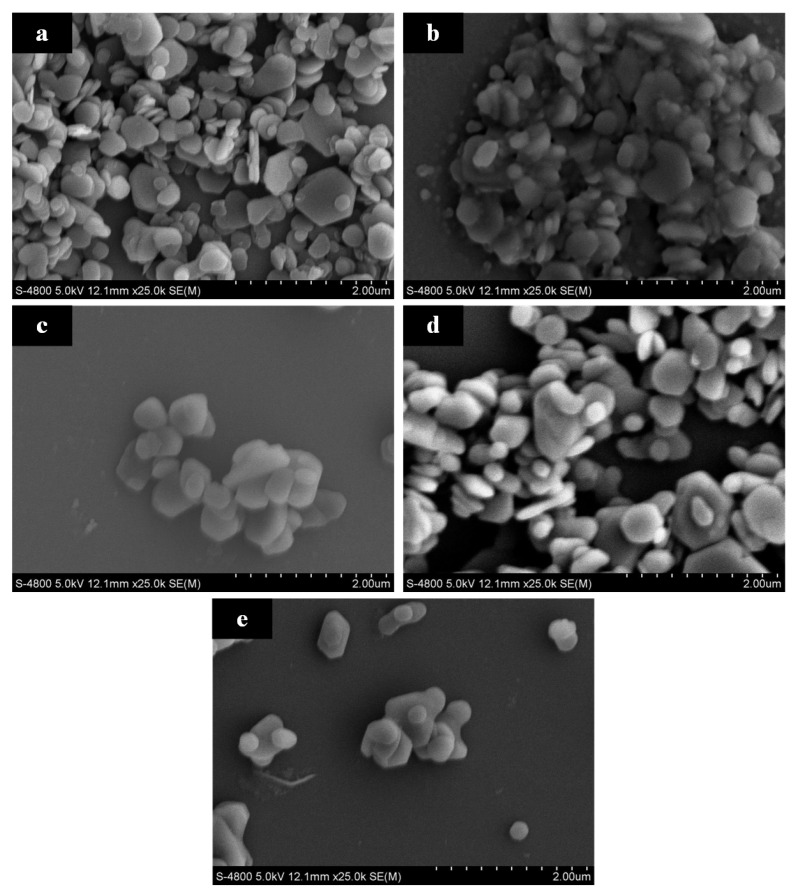
Microscopic morphology of Mg(OH)_2_ crystals at different sampling times: (**a**): 0 min; (**b**): 30 min; (**c**): 60 min; (**d**): 90 min; (**e**): 120 min.

From Figure 11, it can be seen that at 0 min, particles adhere to each other to form clumps, resulting in uneven particle sizes. At 30 min, the particle surfaces appear dissolved, with blurred boundaries and incomplete crystallization, indicating that the particles are in a dissolution-dominant stage. At subsequent timings, the particle boundaries become clear, and the overall appearance is of dispersed particles with improved surface adhesion phenomena.

**Figure 12:**
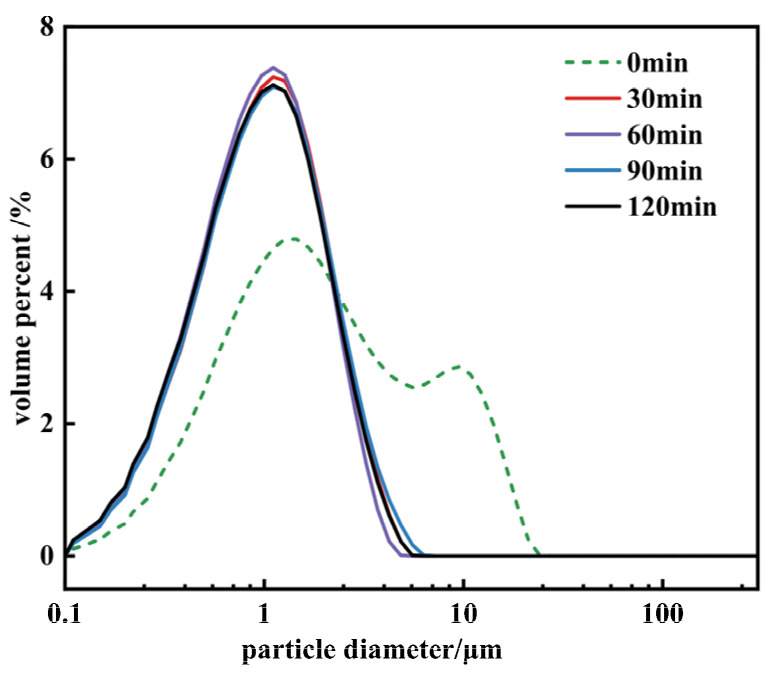
The particle size distribution of Mg(OH)_2_ synthesized at different sampling times.

Figure 12 shows the particle size distribution of Mg(OH)_2_ at different sampling times. The characteristic particle size data of the crystals are listed in Table 1. From Figure 12, at 0 min, the sample exhibits a bimodal distribution in the particle size distribution curve, consistent with the phenomena shown in the SEM images, indicating serious secondary agglomeration and strong particle polarity at this time. Continuing the reaction to 30 min, the characteristic particle size D_90_ decreases from 9.31 μm to 2.17 μm, and D_50_ from 1.69 μm to 0.92 μm, indicating a reduction in the adhesive forces between crystals and a noticeable improvement in the agglomeration issue of the powder on a dry basis. After 60 min, the characteristic particle sizes D_50_ and D_90_ fluctuate slightly around 0.9 μm and 2.1 μm, respectively, suggesting that after 60 min, the change in adhesive forces between crystals is not significant, having a minimal impact on particle agglomeration, with no further improvement in the agglomeration phenomenon.

**Figure 13:**
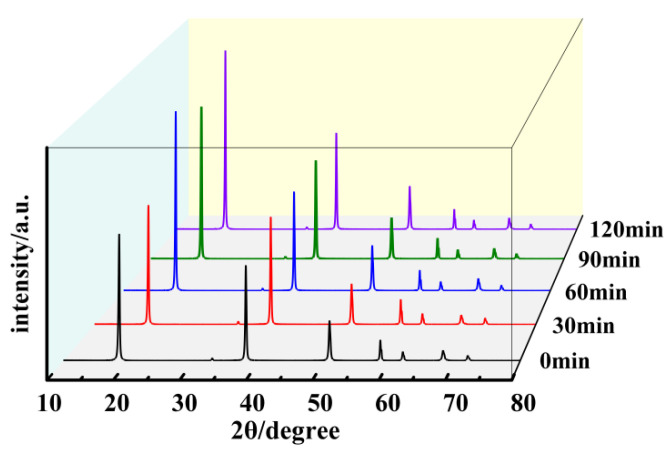
The XRD patterns of Mg(OH)_2_ synthesized at different sampling times.

Figure 13 presents the XRD patterns of Mg(OH)_2_ at different sampling times. The data obtained from XRD are also listed in Table 1. The figure reveals that upon the reaction system reaching 160 °C, all exhibited peaks correspond to characteristic peaks of Mg(OH)_2_ crystals, indicating that magnesium oxide has fully converted into Mg(OH)_2_ at this point, with subsequent times representing the growth phase of Mg(OH)_2_.

Table 1 indicates that within the 0~30 min interval, there is almost no change in the (001) crystallographic plane, while the peak intensity of the (101) crystallographic plane strengthens, indicating improved crystallization in the direction of the (101) crystallographic plane. After 60 min, there is almost no change in the (101) crystallographic plane, but fluctuations are observed in the peak intensity of the (001) crystallographic plane, overall indicating a direction toward improved crystallization. The increase in the exposure level of the crystal faces, resulting in a higher I001/I101 ratio, leads to a reduction in surface polarity and an improvement in the sample’s dispersion. Considering the FWHM values and characteristic particle sizes, the main changes in the crystals occur within the 0~60 min interval, with minor changes thereafter.

Table 2 provides a detailed breakdown of the changes in particle size composition of magnesium hydroxide at different intervals during the reaction process.

Initial Stage: The particle size distribution initially shows a higher percentage of finer particles (<0.2 μm), with moderate to low percentages in larger size ranges.

30 min: Significant dissolution and recrystallization occur, increasing the proportion of the smallest particles (<0.2 μm) and promoting growth into the 0.6–1 μm range.

60 min: The proportion of particles in the <0.2 μm range decreases significantly, indicating a decrease in newly nucleated particles or dissolution of smaller particles, while particles in the 0.2–0.6 μm range see a significant increase, suggesting a shift toward medium-sized particle growth due to crystal stacking and attachment.

Post 60 min: The particle size distribution continues to fluctuate with an overall trend toward larger sizes, indicating ongoing dissolution and recrystallization processes, with an increase in the proportion of larger particles (>1 μm). This evolution pattern suggests that Mg(OH)_2_ particle growth is dynamic, with a notable shift from the formation and dissolution of smaller particles to the growth and stabilization of larger particles as the reaction progresses, particularly moving toward a crystallization dominant phase.

According to the solubility equilibrium constant Formula (3) for Mg(OH)_2_, it is observed that a lower concentration of Mg^2+^ drives the reaction to the right, favoring the hydrolysis of Mg(OH)_2_ and making the dissolution process predominant during the growth phase. When reacting with a higher concentration of MgO, as MgO hydrolyzes, the concentration of Mg^2+^ in the solution increases, aiding in the crystallization and precipitation of Mg(OH)_2_ growth units [33]. At this point, Mg(OH)_2_ is in a coexistence stage of dissolution and crystallization. However, an excessively high concentration of MgO undergoing hydrolysis in the reaction causes the Mg^2+^ concentration in the solution to reach supersaturation, immediately replenishing the Mg^2+^ consumed by the reaction and continuously pushing the reaction to the left, making crystallization the dominant process. This is not conducive to the self-perfection of Mg(OH)_2_ crystals.

(3) $$K_{sp}=c\left[{Mg}^{2+}\right]*{c[{OH}^{-}]}^{2}$$

## 4. Conclusions

This paper uses MgO as the raw material and deionized water as the solvent, conducting hydrothermal treatments under various temperatures, durations, and concentrations to analyze the growth process of Mg(OH)_2_. We establish the significant impacts of hydrothermal temperature, reaction time, and MgO concentration on the crystallization and morphological attributes of Mg(OH)_2_. These factors collectively influence the dissolution–recrystallization dynamics, particle size distribution, and micromorphology, guiding the path toward optimized industrial-scale production. The following conclusions are drawn from analyses conducted through XRD, SEM, and particle size distribution tests:

1. Temperature Impact: The growth morphology and crystallinity of Mg(OH)_2_ are significantly influenced by the hydrothermal temperature. Higher temperatures facilitate the formation of well-defined crystalline structures due to enhanced dissolution and recrystallization processes.
2. Reaction time Effect: Extended hydrothermal durations lead to larger, more uniformly sized Mg(OH)_2_ particles. This suggests that longer reaction times allow for the continuous growth and improvement of crystalline perfection.
3. Concentration Influence: The concentration of MgO plays a critical role in the crystallization process of Mg(OH)_2_. Lower concentrations favor the dissolution process, leading to finer particles, while higher concentrations promote crystallization, resulting in larger crystal sizes. However, excessively high concentrations may lead to supersaturation, hindering the self-perfection of Mg(OH)_2_ crystals by promoting rapid precipitation without sufficient time for orderly crystal growth.
4. Micromorphology Change: SEM images reveal that the particle morphology of Mg(OH)_2_ changes with varying hydrothermal conditions. The transition from irregular agglomerates to more uniform and discrete particles indicates the influence of hydrothermal parameters on particle shape and aggregation behavior.
5. Particle Size Distribution: The particle size distribution shifts toward larger sizes with increased treatment time and temperature, reflecting the growth dynamics of Mg(OH)_2_ particles. The dissolution and recrystallization mechanism is evident from the changing particle size distributions, with initial stages showing a wide distribution that narrows as crystals grow and mature.

These findings offer support for the industrial-scale production of Mg(OH)_2_, providing a foundational understanding for optimizing synthesis conditions to achieve desirable material characteristics efficiently and effectively. By identifying the key parameters that influence the growth and crystallinity of Mg(OH)_2_, this research enables the development of more controlled and scalable hydrothermal synthesis processes, facilitating the broader application of Mg(OH)_2_ in various industries.

## Figures and Tables

**Table 1:** Crystal parameters of Mg(OH)_2_ at different sampling moments.

	I001 Crystallographic Plane			I101 Crystallographic Plane					
	2θ /Degree	Intensity /a.u.	FWHM /Å	2θ /Degree	Intensity /a.u.	FWHM /Å	D_50_ /μm	D_90_ /μm	I001/I101
0 min	18.57	77,997	0.218	37.97	58,735	0.222	1.69	9.31	1.33
30 min	18.57	76,013	0.189	37.97	68,577	0.206	0.92	2.17	1.11
60 min	18.57	118,004	0.178	37.97	65,285	0.199	0.88	2.01	1.81
90 min	18.57	103,544	0.177	37.97	67,176	0.203	0.93	2.25	1.54
120 min	18.57	125,377	0.177	37.97	67,758	0.196	0.90	2.14	1.85

**Table 2:** Particle size composition at different sampling times.

Particle Size Range/μm	Particle Proportion				
	0 min	30 min	60 min	90 min	120 min
0~0.2	8.17%	23.26%	0.00%	1.24%	0.00%
0.2~0.4	62.75%	48.84%	38.89%	49.23%	33.33%
0.4~0.6	25.17%	16.28%	41.67%	38.39%	41.67%
0.6~0.8	3.92%	9.30%	11.11%	8.98%	11.67%
0.8~1	0.00%	2.33%	8.33%	1.24%	10.00%
1~1.2	0.00%	0.00%	0.00%	0.62%	1.67%
1.2~1.4	0.00%	0.00%	0.00%	0.31%	1.67%

## Data Availability

The data presented in this study are available on request from the corresponding author due to privacy reasons.
